# Supplementary material for: Effects of the healthy start randomized intervention on psychological stress and sleep habits among obesity-susceptible healthy weight children and their parents
Source: PLoS One. 2022 Mar 10;17(3):e0264514. doi: 10.1371/journal.pone.0264514 (PMC8912262; doi:10.1371/journal.pone.0264514)
Supplement: S1 Table — (PDF) [file pone.0264514.s001.pdf]

Questions selected and modified from the Swedish version of the Parenting Stress Index<br>Questions on parental stress: *“Which changes have you experienced in your life after you had your child/children?”*	
□ Less sleep<br>□ More sleep<br>□ No change	□ Less work<br>□ More work<br>□ No change
□ Less stress<br>□ More stress<br>□ No change	□ Fewer social gatherings in the home<br>□ More social gatherings in the home<br>□ No change
□ Fewer worries<br>□ More worries<br>□ No change	□ Less joy of life<br>□ More joy of life<br>□ No change
□ Less time to yourself<br>□ More time to yourself<br>□ No change	□ Less everyday surplus energy<br>□ More everyday surplus energy<br>□ No change
□ Fewer household conflicts<br>□ More household conflicts<br>□ No change	□ Less complexity of being a parent compared to expectations<br>□ More complexity of being a parent compared to expectations<br>□ As expected
Other____________________________________	
