# Supplementary material for: Effects of the healthy start randomized intervention on psychological stress and sleep habits among obesity-susceptible healthy weight children and their parents
Source: PLoS One. 2022 Mar 10;17(3):e0264514. doi: 10.1371/journal.pone.0264514 (PMC8912262; doi:10.1371/journal.pone.0264514)
Supplement: S2 Table — (PDF) [file pone.0264514.s002.pdf]

Minimal detectible effects for study outcomes.

**Outcomes**	**Control group mean (SD)**	**Minimal detectable effect**
Duration of sleep (hours)	10.7 (0.6)	0.2
Onset latency (minutes)	19.1 (15.2)	5.0
Total Difficulties score (points)	6.1 (3.8)	1.2
Prosocial Behavior (points)	7.8 (1.8)	0.6
Parental stress index (points)	13.7 (2.3)	0.7

Assuming 80% power, a significance level at 0.05 and a sample size of 307 participants (n1[180], n2[127])
