# Supplementary material for: Effects of the healthy start randomized intervention on psychological stress and sleep habits among obesity-susceptible healthy weight children and their parents
Source: PLoS One. 2022 Mar 10;17(3):e0264514. doi: 10.1371/journal.pone.0264514 (PMC8912262; doi:10.1371/journal.pone.0264514)
Supplement: S3 Table — (PDF) [file pone.0264514.s003.pdf]

Variable	Observations (n)	Missing (n)	Missing %
Municipality of birth	543	0	0
Sex	543	0	0
Baseline BMI z-score	543	0	0
Baseline age	543	0	0
Maternal educational level	314	229	42
Parternal educational level	304	239	44
Physical activity compared to peers	510	33	6
Duration of sleep	504	39	7
Sleep onset latency	506	37	7
SDQ-Difficulties	509	34	6
SDQ-Strengths	509	34	6
Parental Stress Index	472	71	13
Sleep perception	511	32	6
Afternoon sleep	512	31	6
