# Supplementary material for: Effects of the healthy start randomized intervention on psychological stress and sleep habits among obesity-susceptible healthy weight children and their parents
Source: PLoS One. 2022 Mar 10;17(3):e0264514. doi: 10.1371/journal.pone.0264514 (PMC8912262; doi:10.1371/journal.pone.0264514)
Supplement: S4 Table — (PDF) [file pone.0264514.s004.pdf]

Changes in sleep and stress between baseline and follow-up (1.3 years)

	n	Intervention group<br>Mean change (95% CI)	Control group<br>Mean change (95% CI)	P-value
**Duration of sleep (hours)[1]**	296	0.02 (-0.07; 0.11)	0.04 (-0.03; 0.12)	0.64
**Onset latency (minutes)[1]**	301	-1.25 (-3.17; 0.66)	-3.06 (-4.67; -1.45)	0.16
**SDQ Total Difficulties score (points)[1]**	305	0.13 (-0.39; 0.64)	-0.61 (-1.04; -0.17)	0.03
**SDQ Prosocial Behavior (points)[1]**	304	0.34 (0.10; 0.59)	0.31 (0.10; 0.51)	0.82
**Parenting Stress Index (points)[1]**	273	2.24 (1.89; 2.60)	2.07 (1.77; 2.38)	0.47

SDQ: Strengths and Difficulties Questionnaire

[1]: The difference between groups was tested using linear regression modeling. Results presented as mean (95%CI).
