# Supplementary material for: Effects of the healthy start randomized intervention on psychological stress and sleep habits among obesity-susceptible healthy weight children and their parents
Source: PLoS One. 2022 Mar 10;17(3):e0264514. doi: 10.1371/journal.pone.0264514 (PMC8912262; doi:10.1371/journal.pone.0264514)
Supplement: S5 Table — (PDF) [file pone.0264514.s005.pdf]

Baseline outcomes stratified by completers and non-completers

	Completers		Non-completers		
	**n**	**Mean (SD)**	**n**	**Mean (SD)**	**p-value**
**Duration of sleep (hours)**	303	10.71 (0.62)	201	10.78 (0.62)	0.21
**Onset latency (minutes)**	305	18.93 (15.25)	201	17.78 (12.35)	0.37
**SDQ Total Difficulties score (points)**	306	6.35 (3.75)	203	7.25 (4.14)	0.01
**SDQ Prosocial Behavior (points)**	306	7.84 (1.82)	203	7.58 (1.80)	0.11
**Parenting Stress Index (points)**	284	13.62 (2.57)	188	13.46 (2.53)	0.50

SDQ: Strengths and Difficulties Questionnaire
