# Supplementary material for: Effects of the healthy start randomized intervention on psychological stress and sleep habits among obesity-susceptible healthy weight children and their parents
Source: PLoS One. 2022 Mar 10;17(3):e0264514. doi: 10.1371/journal.pone.0264514 (PMC8912262; doi:10.1371/journal.pone.0264514)
Supplement: S1 Appendix — (PDF) [file pone.0264514.s006.pdf]

**Prevention of weight gain among normal weight, high risk, pre-school children – a randomized controlled interventions study.**

## Introduction

The prevalence of obesity is increasing, causing a great need for effective intervention programs (WHO, 2000). However, even if multiple controlled primary intervention studies among children and adults have been conducted during the past 20-30 years, by far, most have been unable to prevent excessive weight gain. A recent Cochrane review showed that <15% of all the published interventions among children had effect (Summerbell et al. 2002), and suggested that a focus on short-term, behavior change was unlikely to be sustainable or effective in impacting on weight status of children. They concluded that the current evidence suggests that many diet and exercise interventions to prevent obesity in children are in fact not effective in preventing excess weight gain.

Recent Danish research suggests that the determinants behind the increase in obesity prevalence are present already in early childhood, and that prevention of obesity therefore has to start early (Rugholm et al. 2005). Furthermore, it is clear that obesity is under both genetic and environmental influence, and that pre- and perinatal factors also play important roles (Newell et al. 2007). In this regard studies (Danielzik et al. 2004) suggests that three sub-groups are at high risk of becoming obese:
*1) Normal weight* children with overweight among their 1$^{st}$ degree relatives
*2) Normal weight* children with high birth weights or
*3) Normal weight* children from socially disadvantaged families (low socioeconomic status based on educational level).

Targeting these groups for intervention may prove more effective than targeting whole populations of children, where intervention effects confined to the high-risk individuals may be diluted. Such targeted approaches have been successfully applied previously in only one study, a Swedish intervention study among young adults from obese families (Eiben et al, 2006). However, the high-risk approach, using individual risk assessment to identify those eligible for intervention, has not previously been applied to children.

## Purpose

The purpose of the present intervention study is to conduct a randomized controlled intervention to prevent excessive weight gain over 2-4 years among *normal weight* 3-5 year children at high risk, defined as the presence of *at least one* or more of the risk factors mentioned above – e.g. high birth weight (> 4000 grams), pre-pregnancy obesity of mother (BMI > 30 kg/m$^2$) or low social class (class V).
The intervention will include dietary guidance to the family of the child, improvement of physical activity and behavioral modification. If the intervention is effective, the study will show that a targeted individual effort may be needed to prevent excessive weight gain among *normal weight* high-risk children.

## Data and methods

In the current intervention study, data on all births between 2004 and 2007 from 5 municipalities in the Copenhagen region (Høje-Taastrup, Glostrup, Gentofte, Brøndby and

Dragør), will be obtained. The participating communities are considered both rural and urban, and were chosen to ensure diversity.
*Data on birth weight and pre-pregnant BMI of the mother* will be obtained from the National birth register at the National Board of Health, which contains information on all births whether at hospital or at home. In the register, the following variables are relevant for the project:

- birth weight and length,
- abdominal and head circumference of the child,
- pre-pregnant weight and height of the mother,
- pregnancy complications,
- inborn malformations,
- multiple birth,
- number at multiple birth,
- length of pregnancy in days,
- mothers and fathers exact ages at time of birth,
- parity,
- preeclampsi,
- general and complicating medical diseases of the mother,
- CPR-numbers for the mother, father and child,
- community address and civil status of the mother,
- whether the parents were living together at the time of birth.

The variables listed above will be used to exclude children with malformations before randomization or be included as potential confounders in the randomization.

*Data on socioeconomic status* (estimated from education) will be obtained from the clinical database "Børns sundhed".

Sampling: To identify the children to be included in the study, information from birth records and the clinical database on all children born in 2004, 2005, 2006 and 2007 in the participating communities will be screened. The children in high risk of developing overweight will then be selected based on the criteria described above, and contact details will be obtained via the CPR-numbers of the children and parents.
In the present study computer driven randomization of the high-risk children to either the intervention or one of the two control samples will secure the allocation. For the children in the intervention sample information on body weight and height, to assess if children are normal weight or not, will be obtained by the nutritionist, who is also responsible for the practical intervention. In control sample 1 information on height and weight will be obtained from those records made in connection with the routine vaccination of children aged 2, 3, 4 and 5 years, by the general practitioners. For the children in control sample 2, information on body weight and height to assess if the children are normal weight or not will be obtained by the nutritionist. If the child is normal weight, the parents will be told that their child is in high risk of becoming overweight. If the child is normal weight, it will be not be eligible for participation.
The early randomization is done in order to minimize contamination. By randomizing at this point in time, and not after attending the first meeting with the nutritionist, only the children in the intervention group will have contact with a nutritionist, and thereby with the intervention itself. Hence, control group 1 will be blinded to the intervention, but will serve as a mirror of the natural development in body weight among normal weight children of

similar age. This design will amplify the possibility to find an effect of the intervention, if present, without biasing the intervention effects. Minimizing bias is also secured by blinding of the final measures of height and weight, as they will be obtained from school health records when the children are around 7 years of age (in-schooling examinations, irrespective of whether the child belongs to the intervention- or the control group).

Sample size: It is estimated that within the 5 municipalities a total of approximately 8700 children born in the period will continue to live in the municipalities until 2011. About 25 % of the children will be at high risk of developing obesity, based on the three criteria (Danielzik et al. 2004), equivalent to approximately 1890 children. These children will be randomized into the intervention sub-sample (n=680), control sub-sample 1 (n=530) and control sub-sample 2 (n=680). It is expected that 2% (n=37) of the children will be twins (Skytthe et al. 2006). Twins will both be allocated to the group the first twin is placed in by the randomization procedure.

*The intervention group:* During March 2009 through February 2011 individuals in the intervention sub-sample will receive an invitation to visit the nutritionist. The oldest children will be enrolled first to assure that the intervention program will start at ages 2-5 years for all children. It is expected that 40 % of the invited families will not show up at the first presentation (n = 272). Moreover, it is expected that up to 10% of the children may already be overweight at presentation with the nutritionist (n = 41). Families of these children will be offered nutrition counseling equivalent to 1 visit, but they will not be a formal part of the study. If the child is normal weight at presentation (50% of the baseline sample, n=367), the family will be further informed about the study and its purposes, and motivated to participate in the study. Information on dietary habits, physical activity and relevant social factors will be obtained. Also, waist circumference and skin folds will be measured at the first meeting, but as there are no cut-off points on these variables for children, no selection will be applied based on these measurements.

*Control group 1:* Data on height and weight from the children in control group 1 will be obtained from journals of the general practitioners in connection with the vaccination program or 2-3 years health examinations (baseline), where about 70% (n=371) of all 2, 3, 4 and 5 year old children are seen (personal communication with Tatjana Hejgaard the National Board of Health). Here, similarly 90% of the group is expected to be normal weight. These children will form the control group (n=334).

*Control group 2:* During March 2009 through February 2010 individuals in the second control group will receive an invitation to visit the nutritionist. The oldest children will be enrolled first to assure that the intervention program will start at ages 2-5 years for all children. It is expected that 40 % of the invited families will not show up at the first presentation (n = 272). Moreover, it is expected that up to 10% of the children may already be overweight at presentation with the nutritionist (n = 41). Families of these children will be offered nutrition counseling equivalent to 1 visit, but they will not be a formal part of the study. If the child is normal weight at presentation (50% of the baseline sample, n=367), the family will be further informed about the study and its purposes, and motivated to participate in the control group. Information on dietary habits, physical activity and relevant social factors will be obtained. Also, waist circumference and skin folds will be measured

at the first meeting, but as there are no cut-off points on these variables for children, no selection will be applied based on these measurements.

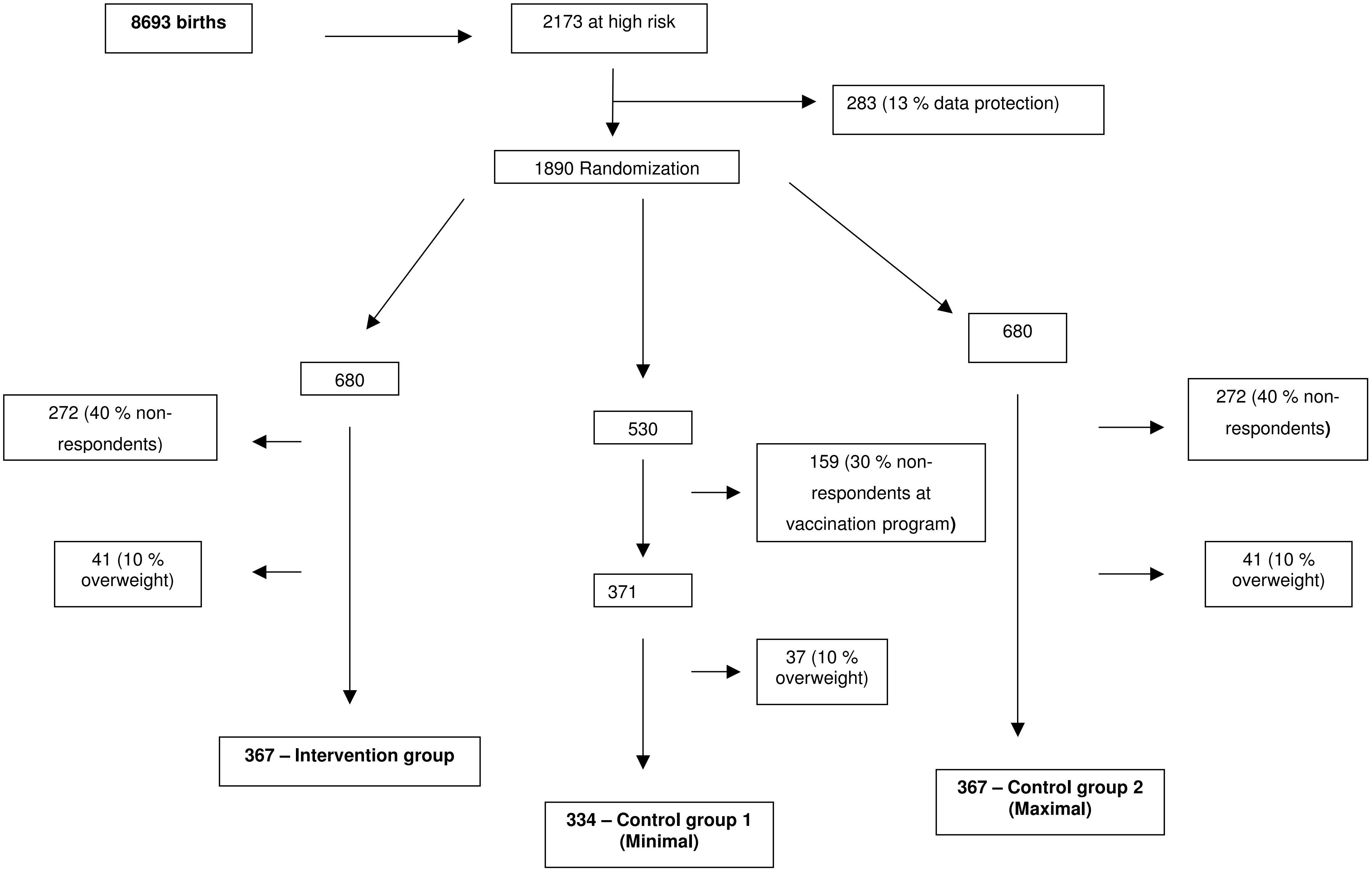
8693 births
2173 at high risk
283 (13 % data protection)
1890 Randomization
680
530
680
272 (40 % non-respondents)
159 (30 % non-respondents at vaccination program)
272 (40 % non-respondents)
41 (10 % overweight)
371
41 (10 % overweight)
37 (10 % overweight)
367 – Intervention group
334 – Control group 1 (Minimal)
367 – Control group 2 (Maximal)

Power calculations: With power of 90% and alpha of 0.05, it will be possible to detect a difference between the intervention group and control group 1 of 0.25 SD. For a 7-year old boy with a height of 1.25 m (equivalent to the mean height for 7-year old children in 1980-1983 (newest data available from school health records)), a 0.25 SD BMI corresponds to BMI=0.40 kg/m$^2$, or 0.63 kg body weight.
With power of 90% and alpha of 0.05, it will be possible to detect a difference between the intervention group and control group 2 of 0.24 SD. For a 7-year old boy with a height of 1.25 m (equivalent to the mean height for 7-year old children in 1980-1983 (newest data available from school health records)), a 0.24 SD BMI corresponds to BMI=0.39 kg/m$^2$, or 0.60 kg body weight.

1) The used non-participation rates could be higher than 40%,
2) With a too small study some of the baseline characteristics could, by chance, be different in the control and intervention group
3) The possibility of making sub-group analysis after the main analysis will have less power, for instance if a potential effect of intervention for the three components of the high risk definition is to be compared.

Intervention: The intervention towards each single family is expected to be in progress for 2 years, but for logistic reasons the project will run for 3 years. A team of 4 nutritionists will make individual dietary counseling for the entire family. Based on current dietary habits of the single family, the nutritionist will provide guidance on healthy dietary choices for each main meal and snack meals. For the counseling process the nutritionists will be applying motivational interviewing. Guidance in healthy dietary choices will include both guidance on how to compose a healthy meal (main and/or snack meal) in general, and how to replace unhealthy products with healthy products. Guidance on how often, and in what amount, high-fat/high-sugar products such as chips, cake, pastry, sweets and soft drinks should be consumed will also be provided. In order to make it easier for the family to change habits the dietary counseling will be based on the existing food culture in each family, including the economic situation, and securing that economy does not become a barrier to compliance of the study.

The nutritionists and an exercise physiologist will also counsel on other aspects of a healthy lifestyle, in particular on how to change into a more physically active lifestyle, where physical activity is integrated into the existing routines, and on how the entire family can work together on being physically active. Furthermore, exercise networks consisting of 6-8 families, as well as family cooking classes on how to cook healthy food, will be established. These teams/classes will also give the families opportunity to create networks. The nutritionists will be assigned offices in the local areas. Each family will have up to 10 sessions of counseling during the 2-year intervention period. Further counseling can be assigned according to the needs of the single family. The second counseling will take place after 1-2 weeks, and thereafter with intervals of 2-3 months. The cooking classes will take place in the evenings and use school kitchens from the 4 communities. The communities participating in the study will make school kitchens available for the study.
A psychologist will furthermore be affiliated with the study for consultancy, if found necessary for the further adherence and participation in the study. The psychologist may

be involved further regarding counseling of families on behavior change. General information on healthy lifestyle, for instance with ideas for healthy lunch packs, inspiration for physical activity and healthy meal recipes will be sent out to the participants via e-mails, to support and inspire the family on their changing habits.

Statistical analyses: It is planned to apply linear regression analyses and adjustment for multiple testing.

**Planned analyses:** Any exclusion or drop out after randomization will be included in the analysis according to the intention-to-treat principle. Differences between

- Intervention- and control group 1
- Intervention- and control group 2,
- Control group 1 and control group 2

for continuous variables will be tested using student t-test. Binary variables will be tested using chi-square test. Linear regression will be used examine the effect of the intervention group on changes in BMI, waist circumference, skin folds and various nutritional variables controlling for potential confounders: birth weight, socioeconomic status of the parents, mother's pre-pregnant BMI, civil status of the mother at time of birth, complications during pregnancy, gestational length in days, complicating medical diseases, mother's medical diseases, smoking habits, abdominal circumference at birth etc. Subgroup analysis on the participating communities will be adjusted for multiple testing.

**Ethical considerations:**
At the start of the intervention the conversation with the participating families will include information about genetic disposition, and how genes and lifestyle interact to create overweight and obesity. The information will also focus on how participation in the study may help to prevent future overweight or obesity among their now normal weight child. Each family will be given scientific and socially acceptable explanations, based on the outcome of the pilot, for their disposition, which they can refer to towards friends and networks in general. These explanations should contribute to reduce the risk of stigmatization, and to preventing the family from experiencing the project as "victim-blaming".
Approval from the Ethical Committee (journal number H-A-2007-0019) has been obtained.

**Perspectives:** The access to registries makes the study possible and less costly than it would be in most other places out of Scandinavia. Danish registry data are of high quality and the design of this intervention is estimated to be less resource demanding than previous interventions.

The present study focuses on obese mothers, only. However, generally predisposition towards obesity of an offspring is independent of which of the first degrees relatives that are obese. Therefore if the intervention is effective, the identification of high risk but yet normal weight children may, in future municipal- or national intervention programs, also be done using school health registries searching for their obese siblings, or potentially draft board examination records in search for obese fathers. Alternatively, health personal (nurses (sundhedsplejersker) and/or family doctors) may, based on their knowledge about families, refer the normal weight, but high-risk children and their families, to intervention.

Furthermore, this study group would be eligible to later follow up through school registries (udskolingsundersøgelserne), and for the boys potentially through the draft board examinations at age 19 years on average (although these may no longer be complete with respect to the most obese), to assess the possible long term effects of the intervention.

**The applicants possibility for carrying out the study according to plan:**
It is estimated that the applicants have optimal possibilities for carrying out the study according to plan, as the study is fully financed and the applicants already have done a great part of the planning. The applicants have been working with research and conducted own studies for several years. In the final planning faces of the study, excursions are made both nationally and internationally to collect experience and inspiration about conducting intervention studies. Finally, the applicants have great access to collegial advice and support.

## Time frame:

| | July-Dec08 | Jan-June09 | July-Dec09 | Jan-June10 | July-Dec10 | Jan-June11 | July-Dec11 | Jan-Jun12 |
|---|---|---|---|---|---|---|---|---|
| Budgetplanning and employment of staff | → | | | | | | | |
| Selecting and rand-omizing children born in 2003,2004,2005, 2006 | → | | | | | | | |
| Data from birth and from community registers on SES | → | | | | | | | |
| Enrollment in the inter-vention, children born 2004 and jan-june 2005 | → | | | | | | | |
| Invitation to nutritionist | | → | | | | | | |
| 1st visit nutritionist 1 | | → | | | | | | |
| Kitchen course 1 | | → | → | → | → | | | |
| Later visits nutritionists 1 | | → | → | → | → | | | |
| 1st visit nutritionist 2 | | | → | | | | | |
| Kitchen course 2 | | | → | → | → | → | | |
| Later visits nutritionists 2 | | | → | → | → | → | | |
| Writing of e-mails | | → | → | → | → | → | → | |
| Data collection | | → | → | → | → | | | |
| Data cleaning | | | | | | → | | |
| Data analyses | | | | | | → | → | → |

| | July-Dec12 | Jan-June13 |
|---|---|---|
| Data analyse | → | → |
| Elaborating results | → | → |

**References:**

Danielzik S, Czerwinski-Mast M, Langnase K, Dilba B, Muller MJ. Parental overweight, socioeconomic status and high birth weight are the major determinants of overweight and obesity in 5-7 y-old children:baseline data of the Kiel Obesity Prevention Study (KOPS). Int J Obes Relat Metab Disord. 2004 Nov;28(11):1494-502

Hansen S, Froberg K, Gronfeldt V, Hasselstrom H, Andersen L. Do Risk Factors for Cardiovascular Disease cluster in 6-7 Year Old Danish Children: the Copenhagen School Child Intervention Study. Accepted in Pediatr. Exerc. Sci. Sept. 2004

Michelsen N, Zachau-Christensen B. Socialpædiatri. I “Nordisk lærebog i pædiatri”
10. udgave, 3. oplag. 1998, Munksgaard

National Task Force on the Prevention and Treatment of Obesity: Overweight, obesity, and health risk. Arch Intern Med 2000;160:898-904

Newell A, Zlot A, Silvey K, Arail K
Adressing the obesity epidemic: A genomics perspective
Prev Chronic Dis. 2007 Apr;4(2):A31. Epub 2007 Mar 15.

Rugholm S, Baker JL, Olsen LW, Schack-Nielsen L, Bua J, Sorensen TI. Stability of the association between birth weight and childhood overweight during the development of the obesity epidemic. Obes Res. 2005 Dec;13(12):2187-94

Skytthe A, Kyvik K, Bathum L, Holm N, Vaupel JW, Christensen K
The Danish Twin Registry in the New Millenium
Twin Research and Human Genetics December 2006 Vol. 9:6:763-771

Summerbell CD, Waters E, Edmunds LD, Kelly S, Brown T, Campbell KJ. Interventions for preventing obesity in children. Cochrane Database Syst Rev. 2002;(2):CD001871

World Health Organisation: Obesity: Preventing and managing the global epidemic. Report of a WHO consultation on Obesity. WHO Techincal Report Series, no. 840, 2000

Eiben G, Lissner L. Health-Hunters – an intervention to prevent overweight and obesity in young high-risk women Int J Obesity, 2006:30:691-6
