# Supplementary material for: Effects of the healthy start randomized intervention on psychological stress and sleep habits among obesity-susceptible healthy weight children and their parents
Source: PLoS One. 2022 Mar 10;17(3):e0264514. doi: 10.1371/journal.pone.0264514 (PMC8912262; doi:10.1371/journal.pone.0264514)
Supplement: Attachment — Submitted filename: Response_EditorComments.docx [file pone.0264514.s008.docx]

Frederiksberg, August 25^th^, 2021

Manuscript ID PONE-D-21-15575

Dear Editor

Thank you for the comments to our manuscript. The comments are included below and our response to each point is marked in italic. In addition to the comments, we have added some minor typographical and linguistic improvements added to the paper.

All changes in the revised paper are marked with tracked changes. A clean revision with no tracked changes is also uploaded. Please note that the page- and line numbers below are based on the clean revision.

We very much appreciate constructive comments and suggestions.

Editor Comments to Author:

1. The data analytic model is not adequately described, while you say you used regression, I don’t know if this was the outcome regressed on treatment status and controlling for baseline, or was it change in the outcome regressed on treatment status? Please document the analysis so anyone could replicate the results. Also, the language around ITT as the analysis using multiple imputation and protocol as the analysis using listwise deletion is somewhat confusing, it might be better to report just one of these (the MI results would be my preference) in the paper and then note the other one.

*The analyses were* *performed using change in the outcome regressed on treatment status. Based on the comment, the Statistical methods section has been rewritten with the aim of improving the description of the data analytical model.*

*Page 11, line 252:* *“To test the effect of the intervention, linear regression models with treatment status included as the explanatory variable and changes in sleep duration, sleep onset latency, SDQ scores or PSI during follow-up included as the response variables were conducted. All models were adjusted for baseline measure of outcome.”*

*Table 2, headline: “Changes in sleep and stress between baseline and follow-up (1.3 years).”*

*Page 11, line 258: “Children who dropped out between baseline and follow-up were included in the analyses according to the intention-to-treat (ITT) principle using multiple imputation. In the multiple imputations, m=10 complete data sets were generated. In each set of data, the missing values were replaced with imputed values, constructed based on predictive distributions for each of the missing values. Each of the completed data sets were analyzed, and the results from the ten analyses were combined to create a single set of estimates that comprised the variability associated with the missing values. The imputations were made using chained equations as implemented in Stata through the commands ice and mim, and based on the variables described above. Imputations were based on allocated group, municipality of birth, baseline BMI z-score, sex, age at baseline, maternal and paternal educational level, perception of the child’s sleep, if the child takes naps during the day, and physical activity compared to children in the same age. When average nighttime sleep duration was used as outcome, imputations were also made on baseline average nighttime sleep duration and SDQ-TD score. When sleep onset latency was used as outcome, imputations were also made on baseline sleep onset latency and SDQ-TD score. When SDQ-TD score was used as outcomes, imputations were also made on baseline SDQ-TD score and average nighttime sleep duration. When SDQ-PSB score was used as outcome, imputations were also made on baseline SDQ-PSB score and average nighttime sleep duration. When PSI score was used as outcome, imputations were also made on baseline PSI score. All statistical tests were two-sided with a significance level at 0.05. Analyses were performed using Stata SE 14 (StataCorp LP, College Station, Texas, USA; www.stata.com).”*

*Page 12, line 280: “Per protocol analyses, removing data from children who dropped out of the intervention before the 1.3 years follow-up examination, were performed as sensitivity analyses. Per protocol analyses are shown in Supporting S3 table. In addition, possible effect modification by sex was examined in all models.”*

*Thank you for the suggestion to report just one of the analyses. We have revised the manuscript and now present only the MI results and have moved the protocol analyses to Supporting S3 table. We have rephrased the results sections accordingly and have deleted any text on the protocol analyses (pages 14-16). We have also removed the results from the protocol analyses from Table 2.*

*In addition, we have rephrased the abstract and the manuscript text:*

*Abstract, line 46: “…. and 95% Confidence Intervals (95% CI) were analyzed using linear regression intention-to-treat (n=543 (intervention group n=271, control group n=272)) analyses.”*

*Abstract, line 51: “Mean change in SDQ-TD was 0.09 points (95% CI -0.57;0.59) in the intervention group, and -0.69 points (95% CI -1.16; -0.23) in the control group (p=0.06).”*

*Page 11, line 252: “To test the effect of the intervention, linear regression models with treatment status included as the explanatory variable and changes in sleep duration, sleep onset latency, SDQ scores or PSI during follow-up included as the response variables were conducted. All models were adjusted for baseline measure of outcome.”*

*Page 12, line 290: “Sample size was n=543”*

2. There appears to be substantial drop out which is greater in the treatment condition than the control condition.  I would find this concerning in any intervention, while MI may partially adjust for this it wouldn’t adjust for systematic dropout (some families were unhappy with the treatment, or the children were doing well and so they didn’t want any more contact). This should be explored somewhat more and mentioned as a possible reason for failure to find effects.

*We have added a table to the manuscript with outcomes stratified by completers and non-completers, showing that non-completers had a statistically significant higher SDQ-TD score than completers, while no other significant differences were observed.*

*Page 12, line 283: “Supporting S4 table shows the specific outcomes stratified by completers and non-completers. Non-completers had a statistically significant higher SDQ-TD score than completers, while no other significant differences were observed.”*

*This has also been addressed in the Discussion section:*

*Page 17, line 340: “The intervention group had a larger drop-out rate than the control group, which may have introduced a systematic drop-out that is not fully adjusted for in the multiple imputations. Even though we only observed a statistically significant difference between completers and non-completers in SDQ-TD score, this cannot be ruled out as a possible reason for failure to find effects.”*

3. I’m doing this review off-line and so I don’t have access to the clinical trials registration, but I will read it for the next round and would hope that initial protocols were followed and any deviations explained.

*Page 6, line 139: “**The intervention period was shortened from the expected 2 years to 1.3 years, because the planning of practicalities related to the conduction of the study and recruitment of participants took longer than anticipated.”*

*The original protocol has been uploaded as Supporting S1 Appendix.*

4. Power analyses are required and to the extent that these were originally done for these outcomes, the original power analyses should be presented. If they were not done for these outcomes then new power analyses are needed, paying particular attention to justifying the effect size that is hypothesized for each of the outcomes given other intervention studies, clinically meaningful effects, and noting the reliability of the outcome measures (measures with low reliability would generally have smaller effect sizes). There should be enough detail in the power analyses that I could replicate the results.

*Page 10, line 247: “The sample size of the Healthy Start Study was established with the main purpose of having enough statistical power to detect a potential effect of the intervention on the primary outcome (body weight). As the present results represent a secondary study, the sample size was not determined for the purpose of these analyses. However, minimal detectible effects are calculated for all outcomes included in the present study (Supporting S2 table).”*

*Page 17, line 352: “Based on the minimal detectable effects from the power calculations for these secondary outcomes, it is considered unlikely that we have missed intervention effects that would be clinically relevant.“*
