# Supplementary material for: Effects of the healthy start randomized intervention on psychological stress and sleep habits among obesity-susceptible healthy weight children and their parents
Source: PLoS One. 2022 Mar 10;17(3):e0264514. doi: 10.1371/journal.pone.0264514 (PMC8912262; doi:10.1371/journal.pone.0264514)
Supplement: Attachment — Submitted filename: ResponseReviewers_Revision2.docx [file pone.0264514.s009.docx]

Frederiksberg, January 20^th^, 2022

Manuscript ID PONE-D-21-15575R1

Dear Editor

On behalf of all authors, I would like to sincerely thank you and the reviewers for the very thorough and constructive review of our manuscript.

The comments are included below, and our response to each point is marked in italic. In addition to the comments, we have added some minor typographical and linguistic improvements to the paper.

All changes in the revised paper are marked with tracked changes. A clean revision with no tracked changes is also uploaded. Please note that the page- and line numbers below are based on the clean revision.

We very much appreciate the constructive comments and suggestions that we have received.

Editor comments

Thank you for submitting your manuscript to PLOS ONE. After careful consideration, we feel that it has merit but does not fully meet PLOS ONE’s publication criteria as it currently stands. Therefore, we invite you to submit a revised version of the manuscript that addresses the points raised during the review process.

I now have reviews for your paper from 4 reviewers, 3 of whom had significant comments. Based on the consensus of these reviewers and my own reading of your paper my editorial decision is that a minor revision is still needed before the paper could be published in Plos ONE. Please pay attention to all of the comments, that said, I disagree with reviewer 1 who says that the analyses on detectable differences are not needed since this is a secondary analysis. While there is some controversy over use of post hoc methods for power, the controversy stems from other issues rather than the issue here where you did not find the effect and are making the case for there being no meaningful effect given the study design. I wouldn’t be willing to accept a paper with null findings without a measure of power, without knowing if you could have found a meaningful effect the results are not very useful. A good reference for this is:

Gelman, A., and Carlin, J. B. (2014). Beyond power calculations: Assessing Type S (sign) and Type M (magnitude) errors. Perspectives on Psychological Science 9, 641-651.

Review 3 notes that it might have been odd to expect an effect on sleep given that most of your subjects were in the normal range to begin with. I think a response to this and inclusion of the power analyses might go hand in hand.

*Thank you very much for the very comprehensive and constructive evaluation of our manuscript. It is greatly appreciated.*

Journal Requirements

Please review your reference list to ensure that it is complete and correct. If you have cited papers that have been retracted, please include the rationale for doing so in the manuscript text, or remove these references and replace them with relevant current references. Any changes to the reference list should be mentioned in the rebuttal letter that accompanies your revised manuscript. If you need to cite a retracted article, indicate the article’s retracted status in the References list and also include a citation and full reference for the retraction notice.

*We have scrutinized the reference list and have not identified any articles that have been retracted.*

*We have made the following changes to the reference list:*

*Reference 1 has been corrected (publication date updated)*

*Reference 14 has been corrected (author list updated (American Academy of Sleep Medicine has been spelled out))*

*Reference 31 in Revision 1 (Knudsen LB, Olsen J. The Danish Medical Birth Registry. Dan Med Bull. 1998;45(3):320-3.) has been replaced with reference 23 in Revision 2 (Bliddal M, Broe A, Pottegard A, Olsen J, Langhoff-Roos J. The Danish Medical Birth Register. Eur J Epidemiol. 2018;33(1):27-36.), as this reference is more new.*

*New references have been added according to editor and reviewer comments (reference numbers 2, 3, 27, 34, 36, 47-54)*

Additional Editor Comments (if provided):

Review Comments to the Author

Please use the space provided to explain your answers to the questions above. You may also include additional comments for the author, including concerns about dual publication, research ethics, or publication ethics. (Please upload your review as an attachment if it exceeds 20,000 characters)

Reviewer #1

Since this is the secondary analysis, it is not meaningful to calculate the detectable effect size based on the collected sample size. Remove the sentence of Line 250-251 and table S2. You can add the reference to the power for the main study in Line 247-249.

*We respectfully disagree with this comment, as the results may not be very useful without knowing if meaningful effects could have been detected.*

*Line 260-261: “…, as results may not be useful without knowing if meaningful effects could be detected”*

Table 1. Since Wilcoxon was used, better present median with IQR.

*Thank you for bringing this to our attention. We did not test for differences in baseline characteristics because of the randomized design. The footnote on using Wilcoxon and Chi-squared tests has therefore been deleted.*

Table 2 footnote, add covariates adjusted in the regression model.

*Table 2 footnote: “Difference between groups tested using linear regression modeling with information on outcome, group allocation, and baseline measure of outcome.”*

Did you test normality of the regression, however, nonparametric method was used in Table 1. This needs justification.

*As described above, the footnote on nonparametric tests has been deleted from Table 1.*

*Model assumptions were assessed for all models through normal probability plots and residual plots. This has been specified:*

*Lines 265-266: “Model assumptions (consistency with a normal distribution and variance homogeneity) were assessed for all models through normal probability plots and residual plots.”*

Flowchart better use two arms for intervention and control groups.

*The flowchart has been updated, using two arms for intervention and control groups*

You can briefly mention the findings from the sensitivity analysis in the Results section.

*Thank you for this great suggestion. We have added the following sentence in the results section:*

*Lines 309-310: “Sensitivity analyses showed essentially similar results as the ITT analyses presented below (Supporting S4 table). “*

Reviewer #2

Dear authors and editorial team,
Thank you for the opportunity to review this manuscript. I declare that I do not have any competing interests in completing this role. The aim of this study was to investigate changes to children’s sleep quality and parents’ and children’s perceived stress as secondary outcomes of a family-based obesity prevention randomized controlled trail. The authors report no statistically significant differences at follow-up, but nevertheless, this research adds to our collective understanding of the many factors that shape children’s obesity and chronic disease risk. The manuscript is well-written and the analyses are thorough. I have provided minor suggestions to improve clarity. Thank you for continuing this important work into protecting families’ long-term health and happiness.

*Thank you for the kind words on our work.*

Introduction, page 3, lines 62-70: The first sentence provides a good opening, but the remainder of the paragraph seems somewhat abrupt. Your abstract introduction presents the link between children's stress and chronic illness risk upfront, and I would recommend adding a similar sentence or two to this beginning paragraph to underscore the relevance of chronic stress to children's health, then continue with the description of stress assessment strategies. E.g., "... chronic exposure to environmental stressors is common. This has adverse implications for children's health because chronic stress is associated with obesity risk, poor sleep, etc.".

*Thank you for this suggestion. We have added the following sentence to the introduction:*

*Lines 60-63: “As chronic stress can initiate inflammatory processes in the body, which can be expressed e.g. in adipose tissue, muscle mass and hormones, stress may have adverse implications for children’s health, and may lead to an increased risk of obesity, metabolic syndrome, and cardiovascular disease”*

Additionally, no capitalization is required after the semicolon in line 64 (“In” to “in”).

*Corrected*

Introduction, page 3, line 79: “suggest” should be “suggests”.

*Corrected*

Child stress methods, page 8, line 196: I believe you meant “100,000 children” with a comma instead of a period.

*Corrected*

Child stress methods, page 9, line 215: capitalize “Swedish”.

*Corrected*

Modified intention-to-treat analyses, page 11, lines 268-275: It would be helpful to the reader to contextualize the missing data if the numbers of participants with imputed values were described. This could be added in line where each variable is described, e.g. “Where PSI score was used as an outcome, imputations were also made on baseline PSI score for n = X participants”.

*Thank you for this suggestion. We have added this information to the manuscript and have made a new Supplemental S3 table.*

*Lines 289-292: “The number of missing values ranged from 0 for the variables allocated group, municipality of birth, baseline BMI z-score, sex, and age at baseline, to 239 for paternal educational level. Distribution of observed and missing values of each variable included in the imputations are shown in Supporting S3 table.”*

Sensitivity analyses, page 12, line 282: What is meant by “possible effect modification by sex”? Was this tested as moderation with interaction terms, in mediation path analyses, or another method you could describe further?

*This has now been specified:*

*Lines 300-302: “In addition, possible effect modification by sex was examined for all outcomes by adding product terms to the models. Subgroup analyses were conducted if statistically significant interactions were observed.”*

*Lines 310-311: “We found no evidence of effect modification by sex for any of the outcomes (all P> 0.102).”*

Discussion, page 19, lines 388-389: I agree that multi-parent responses to the SDQ may have complicated the data. I also wonder if this applies to the PSI results. You mention earlier in the paper that gender of the reporting parent was not assessed, but is it possible to know if the same parent completed both baseline and follow-up PSI surveys? Perceived stress is a highly subjective and gendered concept, and so potential differences between family members or gender differences in stress perception may have also contributed to the observed results. Cooke and colleagues explore this in their paper, albeit for a different family stress scale (DOI: 10.1177/0748175615578756).

*Thank you for making this excellent point. We do not have information on whether the PSI surveys were completed by the same parent at baseline and follow-up. We have added this as a limitation in the discussion section:*

*Lines 415-419: “Likewise, we do not have information on whether the same parent completed the PSI questionnaire at baseline and follow-up. This may have introduced some misclassification, because perceived stress may differ between mothers and fathers, as explored (for a different family stress scale), by Cooke and colleagues. The consequence is an attenuation of the observed differences.”*

Figure 1: Small formatting adjustment needed to read all content in box with “Randomized to allocation group followed in national registers”.

*Thank you for bringing this to our attention. Figure 1 has been updated, including the box of the shadow control group.*

Reviewer #3

Below are my comments:

BACKGROUND/HYPOTHESIS
1. While the references (2&3) supports the level of cortisol reactivity to stress in children predicts internalizing problem in a later age, which illustrates an association between cortisol level and behavioral problem. Nonetheless, behavioral problem in children could be related to a wide range of factors such as parenting skills and child temperament. It does not seem appropriate to assume SDQ is a measurement of child stress. Rather, the authors could make it explicit that this manuscript focuses on the behavioral problems of the participants.

*Thank you for this constructive comment. We have added the point that behavioral problems may not reflect stress as a limitation in the discussion section:*

*Lines 419-423: “**In our study, child behavioral problems were used as an indicator of child psychological stress.* *Even though previous studies have found associations between behavioral problems and cortisol, behavioral problems could also be related to factors such as infant temperament and parenting skills. Another limitation to our study is hence that the extent to which SDQ-TD scores reflect stress in the child remains an open question.”*

*To make it explicit that this manuscript focuses on child behavioral problems, we have made the following adjustments:*

*Lines 95-96: “The aim of this study was to evaluate the effects of a multifaceted intervention on child behavioral problems, parental psychological stress, as well as on sleep duration and sleep onset latency.”*

*Line 193: Headline has been changed to “Child behavioral problems”*

*Line 316: Headline has been changed to “Child behavioral problems”*

*Lines 194-195: “To indicate child psychological stress, the Danish single-sided version of the Strengths and Difficulties Questionnaire was included in the parental questionnaire.”*

2. As commented by the authors, the baseline sleep duration and SDQ are within normal range, what would be the intended intervention effects that the authors would like to see? Would there be a subgroup of participants who had subthreshold sleep and stress problem that could benefit more from the intervention?

*Thank you for this excellent question. Considering that the primary aim of the intervention was to prevent overweight development, and that short sleep duration and a high level of stress are considered risk factors for overweight development, the intended intervention effects that we would have liked to see are a longer sleep duration in the intervention group compared to the control group, and a lower SDQ score in the intervention group compared to the control group. It could certainly be hypothesized that there would be a subgroup of participants who had subthreshold sleep and stress problems, where any larger intervention effects may have been diluted. Unfortunately, we do not believe our sample has sufficient statistical power to conduct such analyses.*

METHOD
1. From the original protocol attached as Appendix 1, the intervention described focused on nutritional counselling and physical activity. Whereas in the introduction, line 97, authors reports that the intervention “focused on improving diet, increasing physical activity, improving sleep duration and quality and reducing psychological stress in the family…”. In that case, the authors would have to elaborate on what intervention was being offered to improve the sleep and psychological stress of the family, so as to allow replication by others.

*Lines 153-155: “The sleep component of the intervention was focused on sleep hygiene, and recommendations for sleep duration. The stress component of the intervention was focused on spending more time as a family.”*

*Lines 161-170: “To support the health consultants, and to ensure homogeneity in the delivery of the intervention, detailed descriptions on how to provide the consultations were developed by the health consultants in collaboration with the project management. The content of each session was selected from evidence-based determinants of the lifestyle habits that the Healthy Start intervention study aimed to optimize. For example, a high media consumption has been associated with a shorter sleep duration, and lowering media consumption could therefore increase the sleep duration. The delivery of the sessions was pilot-tested and trained internally by the health consultants and the project management. Detailed descriptions on how each session was provided have been published previously.”*

2. Line 193: for clarity, the authors should explained what consists of the SDQ-TD and SDQ-PSB score.

*Lines 198-202: “The scores of the “Emotional symptoms”, “Conduct problems”, “Hyperactivity/inattention”, and “Peer relationship problems” scales are summed to a SDQ-TD score, based on a scoring syntax available from the SDQ-webpage, and used as an exposure variable. The SDQ-TD score ranges from 0-40 points, with higher scores being worse.*

*Lines 203-205: “The score of the “Prosocial behavior” scale (SDQ-PSB score) is not incorporated into the SDQ-TD score, as absence of prosocial behaviors differs conceptually from the presence of psychological difficulties”*

3. Line 198: for clarity, the authors should state what condition have been validated to use SDQ as a screening tool.

*This paragraph has been rewritten:*

*Lines 206-209: “The SDQ has been completed for nearly 100,000 children and adolescents in both*

*population studies and clinical samples in the Scandinavian countries. Moreover, in four large-scale Danish cohorts, SDQ has shown to be a useful screening tool for boys and girls across age groups (between 5 and 12 years) and raters (parents and teachers)”*

RESULTS
1. Table 1 showed the baseline characteristics of the participants and this should include all participants as illustrate in the flowchart (N=543). In the current version, Table 1 reports only the results of 307 participants, does it mean that data of the remaining 236 participants were already missing at the baseline? In that case, what data were used to compose Suppl Table 4: Baseline outcomes of completers (N=303) and non-completers (N=201)?

*Thank you for bringing this to our attention. We have updated Table 1 so it now includes all participants as illustrated in the flowchart (n=543)*

DISCUSSION
As the participants in this study has normal sleep and stress parameters to begin with, the intervention from this study is also preventive by nature. The authors may discuss about the trajectory/ emergence on the sleep in child (Williamson A et al J Pediatr 2019), and that this study may not have a long enough follow up to capture the prevention effect. Likewise, the authors may analyze a subgroup of high-risk case with subthreshold sleep problem and see if the intervention effects could be seen.

*Thank you for this excellent point. We have added to the discussion section:*

*Lines 342-346: “Because sleep problems may have different trajectories between infancy and middle childhood, as investigated by Williamson and colleagues, another possible explanation for our results could be that the follow-up period was too short to capture a prevention effect. However, the post hoc power calculations of minimal detectable effects in this study does not suggest that we have missed any clinically relevant intervention effects.”*

Reviewer #4

(No Response)
